# Association of vitamin B2 intake with cognitive performance in older adults: a cross-sectional study

**DOI:** 10.1186/s12967-023-04749-5

**Published:** 2023-11-30

**Authors:** Lingyan Zhou

**Affiliations:** Department of Neurology, Shandong Provincial Hospital Affiliated to Shandong First Medical University, Jinan, Shandong China

**Keywords:** Vitamin B2 intake, Cognitive performance, NHANES

## Abstract

**Background:**

To scrutinize the relationship between vitamin B2 consumption and cognitive function based on the NHANES database.

**Methods:**

This cross-sectional study included eligible older adults from the NHANES 2011–2014. Vitamin B2 intake was determined from dietary interview data for two 24-h periods. Cognitive function was evaluated through the Consortium to Establish a Registry for Alzheimer’s Disease (CERAD), Animal Fluency Test (AFT), and Digit Symbol Substitution Test (DSST). The regression analyses were used to evaluate the association of vitamin B2 intake with cognitive performance. Stratified analyses based on gender, race, and body mass index (BMI) were conducted.

**Results:**

Higher vitamin B2 intake was correlated with higher scores on each test. As compared to the lowest quartile, the highest quartile of vitamin B2 intake was related to a 45.1-fold increase (*P* = 0.004) on the DSST test sores. Moreover, those who were males, non-Hispanic whites, or had a BMI of 18.5 to 30 kg/m^2^ had a stronger relationship between total vitamin B2 consumption and cognitive function.

**Conclusion:**

It's possible that older persons who consume more vitamin B2 have enhanced performance in some areas of cognitive function. To determine the causal link between vitamin B2 consumption and cognitive performance, further long-term research is required.

## Introduction

At present, along with the aggravation of population aging, the proportion of age-related cognitive impairment is increasing [1]. According to a report in 2021, the number of patients suffering from cognitive impairment in the United States will increase from 12.23 million in 2020 to 21.55 million in 2060. In just 40 years, the number of people with cognitive impairment around the world would have almost doubled [2]. Cognitive dysfunction has gradually become one of the most serious social health problems around the world. The early intervention and treatment of cognitive impairment has become one of the important events to be solved urgently [3]. Therefore, it is of great significance to explore the modifiable factors related to cognitive performance to prevent the occurrence of cognitive impairment.

Previous studies have reported that different lifestyles and diets may have a certain effect on the prevention and improvement of cognitive impairment [4–6]. Karen M Beathard, et al. found that participants who consumed more than 1.8 mg/day of vitamin B2 (VB2) performed significantly better on visual perceptual-cognitive performance [7]. A recent study found that an intermediate product of VB2 could ameliorate cognitive impairment and dysfunctional synaptic plasticity in an Alzheimer's disease (AD) mice model [8]. Hyesook Kim, et al. pointed out that total B vitamin intake is linked to cognitive function in older people with AD and mild cognitive impairment (MCI) in Southern Korea [9].

VB2, also called riboflavin (7,8-dimethyl-10-ribityl-isoalloxazine), is a micronutrient essential for maintaining human health [10]. Its water-solubility allows easy absorption in the body. Flavin adenine dinucleotide (FAD) and flavin mononucleotide (FMN) are two crucial forms of VB2 that actively participate in various metabolic pathways [11]. VB2 may impact cognitive function through its key players in a range of redox reactions [12]. Moreover, VB2 plays a vital role in chromatin remodeling, DNA repair, protein folding, and apoptosis [13]. However, the precise mechanisms behind these functions remain unclear. Furthermore, limited research has been conducted on the correlation between VB2 consumption and cognitive function in older individuals to date.

In this study, our hypothesis suggests a potential link between the consumption of VB2 and cognitive function in the elderly population. Analyzing data from the National Health and Nutrition Examination Survey (NHANES) database, which includes a representative sample of individuals aged 60 and above, we explore the connection between VB2 intake and cognitive function.

## Materials and methods

### Study design and subjects

NHANES is a cross-sectional survey conducted by the National Center for Health Statistics (NCHS), which is aimed to investigate the health and nutrition status of the United States (US) by collecting data on demographic characteristics, dietary supplements, physical examination, laboratory tests, and questionnaire information [14–16]. NHANES employs a stratified multistage sampling design to obtain a representative sample of US civilians. All procedures were approved by the NCHS Research Ethics Committee and written informed consent was obtained from participants. Further details about NHANES can be found at www.cdc.gov/nchs/nhanes.html. In the current investigation, we used data from the NHANES 2011–2012 and NHANES 2013–2014 cycles.

**Fig. 1:**
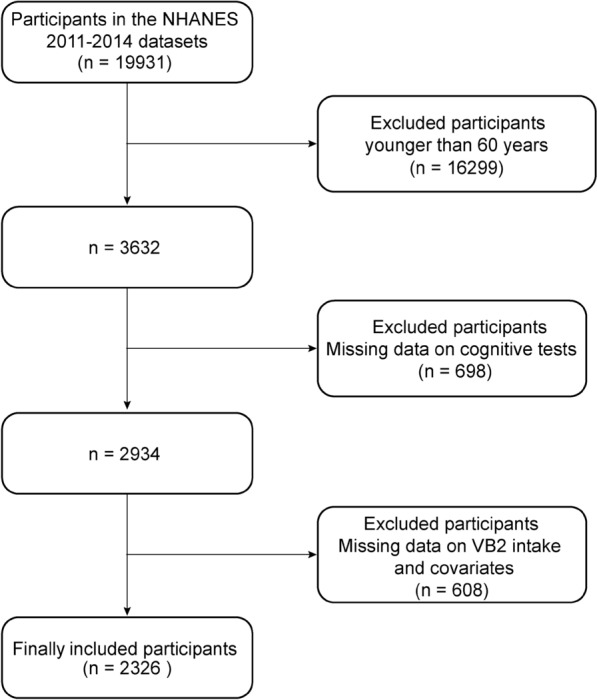
Flow chart of the procedure for the selection of eligible participants

For this cross-sectional analysis, adults aged 60 years or older were included. We excluded missing data on cognitive tests (n = 698), and missing data on VB2 intake and other variables (n = 608). Figure 1 shows the flow of participants throughout the study.

### Total VB2 intake assessment

To estimate intakes of energy, nutrients, and other food components, each NHANES participant supplied full dietary interview data during two 24-h periods. The second dietary recall was obtained by phone 3 to 10 days after the first recall, which was obtained in the Mobile Examination Center [15, 17]. The average of two 24-h dietary recalls was used to assess dietary and supplement intake [18]. The combination of dietary and supplement intake was used to determine total VB2 intake.

### Cognitive function tests

Cognitive function was evaluated through the Consortium to Establish a Registry for Alzheimer’s Disease (CERAD), Animal Fluency Test (AFT), and Digit Symbol Substitution Test (DSST). The CERAD tests are routinely used to assess the immediate and delayed learning ability related to novel verbal information. These inventories consist of three consecutive learning trials (CERAD Trial 1 Recall, CERAD Trial 2 Recall, and CERAD Trial 3 Recall) followed by a delayed recall (CERAD Delayed Recall) [19–21]. The score on each trial ranges from 0 to 10, and the total score of the CERAD test (CERAD total) is the sum of three learning trials. The AFT evaluates executive function by examining categorical verbal fluency with scores from 3 to 39. The DSST constitutes a performance challenge from the Wechsler Adult Intelligence Scale-III that appraises processing speed, sustained attention and working memory, and is scored from 0 and 105 [22]. In each cognitive function test, higher scores indicate better cognitive function [23].

### Covariates

The following covariates are thought to have the potential to cause confounding: age (years), gender (male and female), race (Mexican American, other Hispanic, non-Hispanic White, non-Hispanic Black, and others), family poverty-to-income ratio (PIR), body mass index (BMI, underweight: < 18.5 kg/m^2^; normal: 18.5–25 kg/m^2^; overweight: 25–30 kg/m^2^; obese: ≥ 30 kg/m^2^), total calories (kcal), smoking, drinking (having at least 12 alcohol drinks per year or not). Smoking was defined as never (smoked < 100 cigarettes in life), former (smoking ≥ 100 cigarettes in life but did not smoke currently), and current (smoking ≥ 100 cigarettes in a lifetime and still smoking currently). The levels of alanine aminotransferase (ALT) activity and aspartate aminotransferase (AST) activity in serum were assessed using a kinetic rate technique. The concentration of cholesterol in the serum was determined using the timed-endpoint method.

### Statistical analysis

R software 4.2.3 was used for all data analysis. In our descriptive analysis, categorical variables were expressed as unweighted numbers (n) and weighted percentages (%), and continuous variables were expressed as median (interquartile range, IQR). Total VB2 intake was categorized based on quartiles (Q1: < 25th percentile, Q2: 25 to 50th percentile, Q3: 50 to 75th percentile, Q4: ≥ 75th percentile) with Q1 as the reference category. Wilcoxon rank-sum tests for complex survey samples were employed to calculate the differences in some variables by quartile of total VB2 intake. To find correlations between total VB2 intake and cognitive performance, the univariate and multivariate logistic regression were used to calculate the odds ratio (OR) with 95% confidence interval (CI). Both categorical (quartiles) and continuous factors of total VB2 intake were assessed. Three models were developed: model 1 without covariate adjustment; model 2 adjusted for age; and model 3 adjusted for age, gender, race, PIR, BMI, alcohol intake, and smoking status. Analysis of subgroups was conducted by gender, race, and BMI. Survey design and weighting variables were taken into account in all statistical analyses. Statistical significance was defined as a two-sided *P* < 0.05.

## Results

### Characteristics of participants

A total of 19,931 individuals participated in the NHANES during 2011–2014. After exclusions, our study contained a total of 2326 participants (Fig. 1). The baseline characteristics of all eligible participants were summarized in Table 1. The study cohort had a mean age of 68.00 years. Of those, 53.36% are 60–69 years, 27.04% are 70–79 years, and 19.60% are ≥ 80 years. Study participants were primarily females (53.35%), and non-Hispanic whites (80.23%). 49.65% of participants had never smoked. Alcohol intake was high (73.33%), and overweight (36.39%) or obese was high (37.41%) for the majority of participants. The mean total VB2 intake was 2.54 mg/d. Mean PIR and BMI were 3.18, and 28.10 kg/m^2^, respectively. Total calories were around 1810 kal. Mean ALT, AST, and cholesterol were 20.00 U/L, 23.00 U/L, and 4.89 mmol/L, respectively.

**Table 1:** Characteristics of participants

Characteristic	Overall, n = 2326 (100%)^1^
Gender (n, %)	
Female	1182 (53.35%)
Male	1144 (46.65%)
Age (years)	68.00 (63.00, 74.00)
Age group (n, %)	
60–69 years	1184 (53.36%)
70–79 years	640 (27.04%)
≥ 80 years	502 (19.60%)
Race (n, %)	
Non-Hispanic White	1190 (80.23%)
Non-Hispanic Black	521 (7.89%)
Other Hispanic	225 (3.52%)
Mexican American	200 (3.42%)
Other/multiracial	190 (4.94%)
PIR (%)	3.18 (1.70, 5.00)
Alcohol intake (n, %)	
No	702(26.67%)
Yes	1624 (73.33%)
Smoking status (n, %)	
Never smoker	1134 (49.65%)
Former smoker	899 (39.31%)
Current smoker	293 (11.04%)
BMI (kg/m^2^)	28.10 (24.80, 32.10)
BMI group	
Underweight (< 18.5)	29.00 (1.23%)
Normal (18.5–25)	580.00 (24.97%)
Overweight (25–30)	822.00 (36.39%)
Obese (≥ 30)	895.00 (37.41%)
Total calories (kcal)	1810 (1424.66, 2273.71)
Total VB2 intake (mg)	2.54 (1.70, 3.89)
CERAD Trial 1 Recall	5.00 (4.00, 6.00)
CERAD Trial 2 Recall	7.00 (6.00, 8.00)
CERAD Trial 3 Recall	8.00 (7.00, 9.00)
CERAD total	20.00 (17.00, 23.00)
CERAD Delayed Recall	7.00 (5.00, 8.00)
AFT	18.00 (14.00, 22.00)
DSST	54.00 (42.00, 64.00)
ALT (U/L)	20.00 (16.00, 25.00)
AST (U/L)	23.00 (20.00, 27.00)
Cholesterol (mmol/L)	4.89 (4.14, 5.66)

^1^n (unweighted) (%); Median (IQR)

### The association between total VB2 intake and cognitive performance

As shown in Table 2, we conducted a quartile analysis according to the total intake of VB2. Compared with those with low VB2 intake, participants with higher VB2 intake were more likely to have higher levels of ALT and AST, higher AFT scores, and DSST scores. However, no associations were determined between total VB2 intake and CERAD total score and cholesterol level in the quartile analysis. Table 3 shows the relationship between total VB2 intake as categorical variables and cognitive functions. Compared with Q1, Q4 was found to have better cognitive performance in the CERAD total test, AFT test, and DSST test. The association between the CERAD total test and VB2 intake remained statistically significant in all multivariate logistic regression models after adjusting for several covariates (Table 4). These results reveal that cognitive performance may have a positive association with total VB2 intake.

**Table 2:** Several variables of participants, by quartile of total VB2 intake

Variables	Q1, n = 737 (25%)^1^	Q2, n = 603 (25%)^1^	Q3, n = 528 (25%)^1^	Q4, n = 458 (25%)^1^	*P*^2^
Total VB2 intake	1.29 (1.07, 1.47)	2.06 (1.89, 2.28)	3.18 (2.89, 3.46)	5.26 (4.41, 22.01)	–
AFT	17.00 (13.00, 20.00)	18.00 (14.00, 21.00)	18.00 (14.00, 22.00)	19.00 (15.00, 23.00)	< 0.001
CERAD Trial 1 Recall	5.00 (4.00, 6.00)	5.00 (4.00, 6.00)	5.00 (4.00, 6.00)	5.00 (4.00, 6.00)	0.009
CERAD Trial 2 Recall	7.00 (6.00, 8.00)	7.00 (6.00, 8.00)	7.00 (6.00, 8.00)	7.00 (6.00, 9.00)	0.080
CERAD Trial 3 Recall	8.00 (7.00, 9.00)	8.00 (7.00, 9.00)	8.00 (7.00, 9.00)	8.00 (7.00, 9.00)	0.263
CERAD total	20.00 (17.00, 23.00)	20.00 (17.00, 23.00)	20.00 (17.00, 23.00)	20.00 (18.00, 23.30)	0.098
CERAD Delayed Recall	6.00 (5.00, 8.00)	7.00 (5.00, 8.00)	6.00 (5.00, 8.00)	7.00 (5.00, 8.00)	0.545
DSST	51.97 (37.00, 62.00)	51.00 (42.00, 63.00)	54.00 (42.89, 65.27)	56.00 (47.00, 66.00)	< 0.001
ALT	18.00 (15.00, 24.00)	19.00 (16.00, 25.00)	20.00 (17.00, 26.00)	21.00 (17.00, 26.00)	< 0.001
AST	22.00 (19.00, 27.00)	22.00 (20.00, 27.00)	24.00 (21.00, 27.00)	25.00 (22.00, 28.00)	< 0.001
Cholesterol	4.83 (4.24, 5.77)	4.89 (4.09, 5.60)	4.89 (4.06, 5.70)	4.94 (4.19, 5.66)	0.672

^1^Median (IQR)

^2^Wilcoxon rank-sum test for complex survey samples

**Table 3:** Adjusted OR (95% CI) for scores on CERAD, AFT, and DSST tests across quartile of total VB2 intake

Variables	OR^1^	95% CI^1^	*P*^1^
CERAD Delayed Recall			
Q2 vs Q1	1.30	0.88, 1.91	0.177
Q3 vs Q1	1.23	0.90, 1.69	0.183
Q4 vs Q1	1.39	1.00, 1.92	0.048
CERAD Trial 1 Recall			
Q2 vs Q1	1.30	1.01, 1.68	0.042
Q3 vs Q1	1.32	1.10, 1.60	0.005
Q4 vs Q1	1.38	1.09, 1.75	0.011
CERAD Trial 2 Recall			
Q2 vs Q1	1.20	0.91, 1.59	0.175
Q3 vs Q1	1.08	0.83, 1.42	0.538
Q4 vs Q1	1.50	1.09, 2.06	0.015
CERAD Trial 3 Recall			
Q2 vs Q1	1.14	0.87, 1.49	0.331
Q3 vs Q1	1.19	1.00, 1.43	0.049
Q4 vs Q1	1.15	0.88, 1.50	0.279
CERAD total			
Q2 vs Q1	1.78	0.87, 3.67	0.110
Q3 vs Q1	1.71	1.02, 2.87	0.043
Q4 vs Q1	2.38	1.22, 4.63	0.014
AFT			
Q2 vs Q1	1.01	0.54, 1.89	0.981
Q3 vs Q1	2.19	1.07, 4.46	0.033
Q4 vs Q1	2.79	1.19, 6.51	0.021
DSST			
Q2 vs Q1	1.85	0.29, 11.9	0.495
Q3 vs Q1	7.35	1.21, 44.6	0.032
Q4 vs Q1	45.1	3.99, 510	0.004

^1^adjusted for age, gender, race, PIR, BMI, alcohol intake, and smoking status

**Table 4:** The association between cognitive tests and total VB2 intake

Cognitive tests	OR	95% CI	*P*
CERAD Delayed Recall			
Model 1	1.01	0.99, 1.02	0.338
Model 2	1.01	0.99, 1.02	0.330
Model 3	1.00	0.99, 1.01	0.748
CERAD Trial 1 Recall			
Model 1	1.01	1.00, 1.02	0.025
Model 2	1.01	1.00, 1.02	0.023
Model 3	1.01	1.00, 1.02	0.074
CERAD Trial 2 Recall			
Model 1	1.01	1.01, 1.02	< 0.001
Model 2	1.01	1.01, 1.02	< 0.001
Model 3	1.01	1.00, 1.02	0.002
CERAD Trial 3 Recall			
Model 1	1.01	1.00, 1.02	< 0.001
Model 2	1.01	1.01, 1.02	< 0.001
Model 3	1.01	1.00, 1.01	0.005
CERAD total			
Model 1	1.04	1.02, 1.06	< 0.001
Model 2	1.04	1.02, 1.06	< 0.001
Model 3	1.03	1.01, 1.04	0.003
AFT			
Model 1	1.01	1.0, 1.03	0.149
Model 2	1.01	0.99, 1.04	0.174
Model 3	1.00	0.98, 1.02	0.715
DSST			
Model 1	1.11	1.05, 1.19	0.002
Model 2	1.11	1.04, 1.19	0.004
Model 3	1.03	0.97, 1.09	0.350

Model 1: without covariate adjustment

Model 2: adjusted for age

Model 3: adjusted for age, gender, race, PIR, BMI, alcohol intake, and smoking status

### Stratified analyses by gender, race, and BMI

We also carried out stratified analyses by gender, race, and BMI in the current study. The outcomes are displayed in Tables 5, 6, and 7. We found that there was still a correlation between several cognitive tests and total VB2 intake based on gender, race, or BMI. Due to the small number of individuals with a BMI < 18.5 kg/m^2^, the link between cognitive tests and total VB2 intake was not assessed for these models. These findings demonstrate that among the population of men, non-Hispanic whites, or those with a BMI of 18.5–30 kg/m^2^, CERAD total test was more closely related to total VB2 intake. The results of the stratified study suggest that for certain people, the relationship between cognitive function and total VB2 intake might be stronger.

**Table 5:** The association between cognitive performance and total VB2 intake stratified by gender

Cognitive tests	OR^1^	95% CI^1^	*P*^1^
CERAD Delayed Recall			
Male	1.00	0.97, 1.03	0.993
Female	1.00	0.99, 1.02	0.698
CERAD Trial 1 Recall			
Male	1.02	1.00, 1.04	0.054
Female	1.00	0.99, 1.02	0.677
CERAD Trial 2 Recall			
Male	1.02	1.01, 1.04	0.001
Female	1.00	1.00, 1.01	0.211
CERAD Trial 3 Recall			
Male	1.01	1.00, 1.03	0.083
Male	1.01	1.00, 1.03	0.083
CERAD total			
Male	1.06	1.01, 1.11	0.015
Female	1.01	0.99, 1.04	0.246
AFT			
Male	1.00	0.95, 1.05	0.981
Female	1.00	0.97, 1.02	0.682
DSST			
Male	1.07	0.95, 1.20	0.244
Female	1.01	0.93, 1.09	0.804

^1^adjusted for age, race, PIR, BMI, alcohol intake, and smoking status

**Table 6:** The association between cognitive performance and total VB2 intake stratified by race

Cognitive tests		OR^1^	95% CI^1^	*P*^1^
CERAD Delayed Recall	Non-Hispanic White	1.00	0.99, 1.01	0.762
	Non-Hispanic Black	0.99	0.96, 1.02	0.561
	Other Hispanic	1.01	0.98, 1.04	0.405
	Mexican American	1.06	0.90, 1.25	0.377
	Other/multiracial	1.00	0.96, 1.04	0.864
CERAD Trial 1 Recall	Non-Hispanic White	1.01	1.00, 1.02	0.089
	Non-Hispanic Black	0.98	0.96, 1.00	0.050
	Other Hispanic	1.00	0.98, 1.01	0.733
	Mexican American	1.01	0.91, 1.12	0.836
	Other/multiracial	1.01	1.00, 1.02	0.039
CERAD Trial 2 Recall	Non-Hispanic White	1.01	1.00, 1.02	0.003
	Non-Hispanic Black	1.00	0.96, 1.03	0.780
	Other Hispanic	1.01	0.97, 1.04	0.643
	Mexican American	1.04	0.92, 1.17	0.444
	Other/multiracial	1.02	1.00, 1.04	0.016
CERAD Trial 3 Recall	Non-Hispanic White	1.01	1.00, 1.01	0.008
	Non-Hispanic Black	0.98	0.95, 1.02	0.341
	Other Hispanic	0.99	0.97, 1.02	0.543
	Mexican American	1.03	0.92, 1.14	0.552
	Other/multiracial	1.01	1.00, 1.03	0.090
CERAD total	Non-Hispanic White	1.03	1.01, 1.04	0.004
	Non-Hispanic Black	0.96	0.88, 1.04	0.296
	Other Hispanic	1.00	0.93, 1.07	0.967
	Mexican American	1.07	0.80, 1.44	0.553
	Other/multiracial	1.04	1.01, 1.08	0.007
AFT	Non-Hispanic White	0.99	0.97, 1.01	0.495
	Non-Hispanic Black	1.01	0.89, 1.14	0.919
	Other Hispanic	1.02	0.96, 1.08	0.545
	Mexican American	1.23	1.00, 1.52	0.053
	Other/multiracial	1.02	0.97, 1.08	0.385
DSST	Non-Hispanic White	1.02	0.96, 1.09	0.473
	Non-Hispanic Black	1.12	0.84, 1.48	0.432
	Other Hispanic	1.28	1.05, 1.56	0.019
	Mexican American	1.76	0.97, 3.21	0.060
	Other/multiracial	1.0	0.78, 1.26	0.962

^1^adjusted for age, gender, PIR, BMI, alcohol intake, and smoking status

**Table 7:** The association between cognitive performance and total VB2 intake stratified by BMI group

Cognitive tests	OR^1^	95% CI^1^	*P*^1^
CERAD Delayed Recall			
Underweight (< 18.5)	-	–	–
Normal (18.5–25)	1.01	0.99, 1.02	0.468
Overweight (25–30)	1.01	0.98, 1.03	0.601
Obese (≥ 30)	0.99	0.97, 1.01	0.283
CERAD Trial 1 Recall			
Underweight (< 18.5)	–	–	–
Normal (18.5–25)	1.02	1.00, 1.03	0.028
Overweight (25–30)	1.01	0.98, 1.03	0.526
Obese (≥ 30)	1.00	0.98, 1.02	0.965
CERAD Trial 2 Recall			
Underweight (< 18.5)	–	–	–
Normal (18.5–25)	1.02	1.00, 1.03	0.029
Overweight (25–30)	1.01	0.99, 1.02	0.347
Obese (≥ 30)	1.01	1.00, 1.02	0.117
CERAD Trial 3 Recall			
Underweight (< 18.5)	–	–	–
Normal (18.5–25)	1.01	1.00, 1.02	0.003
Overweight (25–30)	1.01	1.00, 1.02	0.027
Obese (≥ 30)	1.00	0.98, 1.01	0.502
CERAD total			
Underweight (< 18.5)	–	–	–
Normal (18.5–25)	1.04	1.01, 1.07	0.005
Overweight (25–30)	1.03	0.98, 1.07	0.242
Obese (≥ 30)	1.00	0.97, 1.04	0.758
AFT			
Underweight (< 18.5)	–	–	–
Normal (18.5–25)	1.00	0.97, 1.03	0.805
Overweight (25–30)	1.03	0.98, 1.07	0.254
Obese (≥ 30)	0.96	0.93, 0.99	0.006
DSST			
Underweight (< 18.5)	–	–	–
Normal (18.5–25)	1.01	0.91, 1.13	0.834
Overweight (25–30)	1.03	0.93, 1.15	0.529
Obese (≥ 30)	1.04	0.95, 1.14	0.382

^1^adjusted for age, gender, PIR, race, alcohol intake, and smoking status

## Discussion

This analysis used data from two cycles of NHANES (2011–2012, 2013–2014) to examine the links between VB2 intake and cognitive function among older adults in the United States. We found that higher VB2 intake was connected to better cognitive performance. For example, compared with the lowest quartile, the highest quartile of VB2 intake was associated with a 45.1-fold increase on the DSST test scores. Additionally, male, non-Hispanic white, or individuals with a BMI of 18.5–30 kg/m^2^ might have a stronger correlation between total VB2 intake and cognitive function.

The relationship between VB2 consumption and cognitive function or dementia has been examined in several epidemiological studies. Atsushi Araki and colleagues discovered that consuming sufficient amounts of VB2 could aid in preventing cognitive deterioration among older men diagnosed with diabetes mellitus [24]. Hai Duc Nguyen, et al. found that increasing daily intake of a combination of B vitamins (VB1, VB2, VB3, VB6, VB9, VB12) might contribute to reducing the risk of dementia [25]. Yeneisy Lanyau-Domínguez, et al. observed that VB2 deficiency was associated with AD and MCI [26]. A prospective cohort study demonstrated that higher intake of VB2 could improve cognitive performance across multiple domains in middle-aged and elderly individuals [27]. One study conducted on a Chinese population residing in Singapore revealed that individuals who consumed higher levels of VB2 during their middle age experienced a reduced risk of cognitive decline in their later years [28]. Our study also corroborates these previous researches, demonstrating a positive relationship between increased VB2 intake and enhanced cognitive performance. On the contrary, a study conducted by G McNeill, et al. failed to find any substantial evidence supporting the beneficial impact of B vitamin intake on cognitive function among individuals aged 70 and above [29]. A recent randomized controlled trial concluded that short-term VB2 supplementation in healthy children had no noticeable effect on cognitive performance [30]. In addition, several studies have explored the relationship between other dietary intake and cognitive performance. Kai Zhang, et al. demonstrated that the folate intakes were inversely associated with low cognitive performance [31]. Meanwhile, Suyun, Li et al. revealed that the prevalence of low cognitive performance may be inversely associated with the intake of total zinc, copper, and selenium [32]. Combined with our findings, all the available evidence suggests that dietary modifications may have potential preventive and ameliorative effects on cognitive impairment. Further studies are warranted to confirm a definitive association.

It has been reported that VB2 possesses antioxidant, anti-inflammatory, and anti-aging properties [33–35]. Previous studies indicate that VB2 reduces oxidative stress and oxidative DNA damage in diabetic mice [12] and relieves shock caused by liposaccharide [36]. Moreover, VB2 alleviates hepatocellular injury and subsequent liver ischemia/reperfusion injury through promoting antioxidation [37]. In mouse models, VB2 deficiency disrupts proper mitochondrial development and impairs mitochondrial function in the liver, while VB2 supplementation improves mitochondrial function [38]. The study conducted by O Korede, et al. revealed a noteworthy increase in erythrocyte ALT activity following various water-soluble vitamin supplements for a duration of 6 weeks [39]. Hai-Rui Yu, et al. found that ALT and AST activities were significantly decreased with the increase in dietary VB2 level up to 31.81 mg/kg, and then increased with further increased dietary VB2 level. Serum total cholesterol levels were significantly decreased with the dietary of VB2 levels up to 31.81 mg/kg [40]. Our study discovered that participants with higher VB2 intake had higher levels of ALT and AST activities. However, no associations were determined between VB2 intake and cholesterol level. We expect to further investigate this issue to clarify their relationships in the future.

According to a study, more than 65% of individuals who develop late-onset cognitive impairments are women [41]. In fact, gender is one of the strongest risk factors for developing cognitive impairments [41]. A study indicates that Hispanic and African-American populations have a higher risk of dementia compared to the non-Hispanic white population [42]. Having a higher BMI before age 65 (midlife) is linked to poorer cognitive performance, accelerated cognitive decline, and an increased likelihood of cognitive impairment in late life [43]. Based on the factors related to cognitive function in the previous studies, we selected various variables (gender, race, and BMI) and tried to carry out stratified analyses. The findings demonstrate that among the population of men, non-Hispanic whites, or those with a BMI of 18.5–30 kg/m^2^, CERAD total test was more closely related to total VB2 intake. The results of the stratified analyses suggest that for certain people, the relationship between cognitive function and total VB2 intake might be stronger. Further cohort analyses with larger samples are needed to validate these findings.

Large, nationally representative datasets that have undergone meticulous quality control were employed in our study. To correctly evaluate the relationship between VB2 intake and cognitive function in older persons, various confounders were controlled in our analysis. However, it is important to note that our study had several limitations. Firstly, this study is a cross-sectional and observational study based on the NHANES database. Therefore, this study cannot accurately know the causal relationship between VB2 and cognitive function. Secondly, since the dietary data were obtained through questionnaires, there might be some bias or even errors in some food intake. Furthermore, the dietary data is obtained from a 24-h questionnaire and thus could not represent long-term dietary data for the participants. Lastly, because there are relatively few datasets including all three cognitive function tests in the NHANES database, the number of datasets included in this study is limited. Further research is necessary in the future if more datasets are usable.

## Conclusions

In conclusion, our findings suggest that participants who had higher intake of VB2 are associated with better cognitive function. In particular, among the population of men, non-Hispanic whites, or those with a BMI of 18.5–30 kg/m^2^, CERAD total test is more closely related to total VB2 intake. Taken together, our findings indicate that VB2 may potentially reduce the risk of cognitive impairment. Further exploration, such as long-term clinical trials of VB2 supplements, are warranted to confirm the present results and to test the optimal intake of VB2 for older adults to prevent cognitive decline.

## Data Availability

The datasets supporting the conclusions of this study are available from the NHANES database (https://www.cdc.gov/nchs/nhanes/nhanes).

## References

[1] Wang H, Lv Y, Ti G, Ren G (2022). Association of low-carbohydrate-diet score and cognitive performance in older adults: National Health and Nutrition Examination Survey (NHANES). BMC Geriatr.

[2] Rajan KB (2021). Population estimate of people with clinical Alzheimer's disease and mild cognitive impairment in the United States (2020–2060). Alzheimers Dement.

[3] Jeremic D, Jimenez-Diaz L, Navarro-Lopez JD (2023). Targeting epigenetics: a novel promise for Alzheimer's disease treatment. Ageing Res Rev.

[4] Ye KX (2023). The role of lifestyle factors in cognitive health and dementia in oldest-old: a systematic review. Neurosci Biobehav Rev.

[5] McGrattan AM (2019). Diet and inflammation in cognitive ageing and Alzheimer's disease. Curr Nutr Rep.

[6] Kivipelto M, Mangialasche F, Ngandu T (2018). Lifestyle interventions to prevent cognitive impairment, dementia and Alzheimer disease. Nat Rev Neurol.

[7] Beathard KM (2023). The impact of nutrition on visual cognitive performance in the nutrition, vision, and cognition in sport study. Front Nutr.

[8] Kim H, Kim G, Jang W, Kim SY, Chang N (2014). Association between intake of B vitamins and cognitive function in elderly Koreans with cognitive impairment. Nutr J.

[9] Zhang M (2023). Biomimetic remodeling of microglial riboflavin metabolism ameliorates cognitive impairment by modulating neuroinflammation. Adv Sci.

[10] Mazur-Bialy AI, Buchala B, Plytycz B (2013). Riboflavin deprivation inhibits macrophage viability and activity—a study on the RAW 264.7 cell line. Br J Nutr.

[11] Lienhart WD, Gudipati V, Macheroux P (2013). The human flavoproteome. Arch Biochem Biophys.

[12] Alam MM, Iqbal S, Naseem I (2015). Ameliorative effect of riboflavin on hyperglycemia, oxidative stress and DNA damage in type-2 diabetic mice: Mechanistic and therapeutic strategies. Arch Biochem Biophys.

[13] Joosten V, van Berkel WJ (2007). Flavoenzymes. Curr Opin Chem Biol.

[14] Elnakib S (2022). A novel score for mhealth apps to predict and prevent mortality: further validation and adaptation to the us population using the us national health and nutrition examination survey data set. J Med Internet Res.

[15] Saint-Maurice PF (2022). Estimated number of deaths prevented through increased physical activity among US Adults. JAMA Intern Med.

[16] Kim S, Cho J, Shin DW, Jeong SM, Kang D (2023). Racial differences in long-term social, physical, and psychological health among adolescent and young adult cancer survivors. BMC Med.

[17] Jayanama K (2021). Relationship between diet quality scores and the risk of frailty and mortality in adults across a wide age spectrum. BMC Med.

[18] Ued FV (2019). Vitamin B2 and folate concentrations are associated with ARA, EPA and DHA fatty acids in red blood cells of brazilian children and adolescents. Nutrients.

[19] Shi Y (2023). Association between exposure to phenols and parabens and cognitive function in older adults in the United States: a cross-sectional study. Sci Total Environ.

[20] Jaeger J (2018). Digit symbol substitution test: the case for sensitivity over specificity in neuropsychological testing. J Clin Psychopharmacol.

[21] Fillenbaum GG (2008). Consortium to establish a registry for Alzheimer's disease (CERAD): the first twenty years. Alzheimers Dement.

[22] Prokopidis K, Giannos P, Ispoglou T, Witard OC, Isanejad M (2022). Dietary fiber intake is associated with cognitive function in older adults: data from the national health and nutrition examination survey. Am J Med.

[23] Tang H, Zhang X, Luo N, Huang J, Zhu Y (2023). Association of dietary live microbes and non-dietary prebiotic/probiotic intake with cognitive function in older adults: evidence from NHANES. J Gerontol A Biol Sci Med Sci.

[24] Araki A (2017). Low intakes of carotene, vitamin B(2), pantothenate and calcium predict cognitive decline among elderly patients with diabetes mellitus: The Japanese Elderly Diabetes Intervention Trial. Geriatr Gerontol Int.

[25] Nguyen HD, Kim MS (2022). The role of mixed B vitamin intakes on cognitive performance: Modeling, genes and miRNAs involved. J Psychiatr Res.

[26] Lanyau-Dominguez Y (2020). Levels of vitamins and homocysteine in older adults with alzheimer disease or mild cognitive impairment in cuba. MEDICC Rev.

[27] Tao L (2019). Dietary intake of riboflavin and unsaturated fatty acid can improve the multi-domain cognitive function in middle-aged and elderly populations: a 2-year prospective cohort study. Front Aging Neurosci.

[28] Sheng LT (2020). Association between dietary intakes of B vitamins in midlife and cognitive impairment in late-life: the Singapore Chinese health study. J Gerontol A Biol Sci Med Sci.

[29] McNeill G (2011). Antioxidant and B vitamin intake in relation to cognitive function in later life in the Lothian Birth Cohort 1936. Eur J Clin Nutr.

[30] Rauh-Pfeiffer A (2014). Three-month B vitamin supplementation in pre-school children affects folate status and homocysteine, but not cognitive performance. Eur J Nutr.

[31] Zhang K (2023). Association between dietary folate intake and cognitive impairment in older US adults: National Health and Nutrition Examination Survey. Arch Gerontol Geriatr.

[32] Li S, Sun W, Zhang D (2019). Association of zinc, iron, copper, and selenium intakes with low cognitive performance in older adults: a cross-sectional study from national health and nutrition examination survey (NHANES). J Alzheimers Dis.

[33] Plantone D, Pardini M, Rinaldi G (2021). Riboflavin in neurological diseases: a narrative review. Clin Drug Investig.

[34] Ashoori M, Saedisomeolia A (2014). Riboflavin (vitamin B(2)) and oxidative stress: a review. Br J Nutr.

[35] Suwannasom N, Kao I, Pruss A, Georgieva R, Baumler H (2020). Riboflavin: The Health Benefits of a Forgotten Natural Vitamin. Int J Mol Sci.

[36] Shih CK (2010). Riboflavin protects mice against liposaccharide-induced shock through expression of heat shock protein 25. Food Chem Toxicol.

[37] Sanches SC (2014). Riboflavin (vitamin B-2) reduces hepatocellular injury following liver ischaemia and reperfusion in mice. Food Chem Toxicol.

[38] Hoppel CL, Tandler B (1975). Riboflavin and mouse hepatic cell structure and function. Mitochondrial oxidative metabolism in severe deficiency states. J Nutr.

[39] Korede O (1991). Effect of supplementation with water-soluble vitamins on erythrocyte alanine aminotransferase activity of healthy adolescents. Ann Nutr Metab.

[40] Yu HR (2022). Effects of dietary riboflavin supplementation on the growth performance, body composition and anti-oxidative capacity of coho Salmon (*Oncorhynchus kisutch*) post-smolts. Animals (Basel).

[41] Nebel RA (2018). Understanding the impact of sex and gender in Alzheimer's disease: a call to action. Alzheimers Dement.

[42] Alzheimer's A (2014). 2014 Alzheimer's disease facts and figures. Alzheimers Dement.

[43] Crane BM, Nichols E, Carlson MC, Deal JA, Gross AL (2023). Body mass index and cognition: associations across mid- to late life and gender differences. J Gerontol A Biol Sci Med Sci.

